# Non-human Primate Determinants of Natural Killer Cells in Tissues at Steady-State and During Simian Immunodeficiency Virus Infection

**DOI:** 10.3389/fimmu.2020.02134

**Published:** 2020-09-10

**Authors:** Nicolas Huot, Philippe Rascle, Caroline Petitdemange, Vanessa Contreras, Jean-Louis Palgen, Christiane Stahl-Hennig, Roger Le Grand, Anne-Sophie Beignon, Beatrice Jacquelin, Michaela Müller-Trutwin

**Affiliations:** ^1^Unité HIV, Inflammation et Persistance, Institut Pasteur, Paris, France; ^2^Université Paris Diderot, Sorbonne Paris Cité, Paris, France; ^3^CEA-Université Paris Saclay-Inserm, U1184, Center for Immunology of Viral, Auto-immune, Hematological and Bacterial Diseases, IMVA-HB/IDMIT, Fontenay-aux-Roses, France; ^4^Deutsches Primatenzentrum, Göttingen, Germany

**Keywords:** SIV, HIV, NK cells, lymph node, gut, tissue resident, CXCR5, NKp44

## Abstract

Natural killer (NK) cells play essential roles in immunity to viruses and tumors. Their function is genetically determined but also modulated by environmental factors. The distribution and functional regulation of these cells vary depending on the tissue. NK cell behavior in lymphoid tissues is so far understudied. Non-human primate (NHP) models are essential for the development of therapies and vaccines against human diseases, and access to NHP tissues allows insights into spatial regulations of NK cells. Here, we investigated tissue-specific parameters of NK cells from NHP species, i.e., cynomolgus macaque (*Macaca fascicularis*), African green monkey (*Chlorocebus sabaeus*), rhesus macaque (*Macaca mulatta*), and baboon (*Papio anubis*). By comprehensive multi-dimensional analysis of NK cells from secondary lymphoid organs, intestinal mucosa, liver, and blood, we identified tissue- and species-specific patterns of NK cell frequencies, phenotypes, and potential activity. Also, we defined the tissue-specific characteristics of NK cells during infection by the simian immunodeficiency virus. Altogether, our results provide a comprehensive anatomic analysis of NK cells in different tissues of primates at steady-state and during a viral infection.

## Introduction

In recent years, it has become increasingly clear that human natural killer cells (NK cells) are far more diverse (1). Some of the diversity is genetically determined, whereas substantial diversification seems to be also determined by environmental factors, including vaccinations and age (1–4). NK cells are frequent in organs such as the liver and the uterus but are probably present in all tissues to various degrees, including bone marrow, lymph nodes, spleen, kidneys, skin, lung, and gut (2, 5–9). NK cells in secondary and tertiary lymphoid organs are still understudied, given their low frequencies in these lymphoid tissues (10–12). A proportion of them express molecules known as tissue-resident markers for T cells, such as CD69 and CD103 (5). The local tissue environment impacts NK cell diversity and function (5, 13). The role of tissue localization in human NK cell development and function and how circulating NK cells relate to those in different sites are, however, not yet sufficiently well understood. Also, markers for the identification of distinct tissue-resident NK cell subpopulations are still scarce. Characterization of NK cells is particularly challenging in tissues, mainly due to lack of hallmark lineage markers for tissue-derived innate lymphoid cells (ILCs) and NK cells in humans. In the context of viral infections, access limitations to organs in humans are an additional hurdle to fully understand the role of NK cells in tissues.

Non-human primates (NHPs) are essential models for the development of therapies and vaccines against human diseases and allow insights into spatial cellular regulations of NK cells. African green monkeys (AGMs), macaques, and baboons are the most largely used NHP models in pre-clinical research. They have contributed essentially to the research or development of vaccines against viral diseases, including severe acute respiratory syndrome coronavirus 2, HIV, influenza, yellow fever, and Ebola virus (14–18). Efforts to identify subpopulations of NK cells in NHP were complicated by incomplete definitions of them, mainly because the antibody against CD56 does not stain all NK cell subpopulations in NHP species (19). Alternatively, NHP NK cells in the blood have been defined as CD3^–^CD8αα^+^CD20^–/dim^NKG2a/c^+^, which is considered the most effective inclusive definition for NK cells from Old World monkeys, such as rhesus and pig-tailed macaques, sooty mangabeys, and AGMs (20–23), whereas this definition does not fully characterize neotropical primate blood NK cells (24).

Some of the NK cell diversity driven by environmental factors is linked to their maturation and differentiation. In humans, the modulation of NKG2a/c expression among CD56dim cells defines a crucial step of their differentiation (25, 26). Immature and mature NK cells can be respectively defined as CD56^bright^NKG2a/c^hi^CD62L^hi^CD57^–^ and CD56^dim^NKG2a/c^–^CD62L^–^CD57^+^. Their distribution varies according to the tissue. NK cells in tissues also share several markers with ILCs. The natural cytotoxicity receptors (NCRs) NKp46 and NKp44 have been described on both NK cells and ILCs (27). NKp46 is a major determinant of NK cell function and is implicated in tumor immune surveillance in acute myeloid leukemia (28, 29). NKp44 is expressed on activated human NK cells and is important for tumor target cell recognition by NK cells during natural cytotoxicity responses, whereas, in the adult intestine, NKp44 + ILCs are the main ILC subset producing IL-22 (30, 31). NKp44 + CD103 + CD69 + NK cells have been shown to persist in adult intestine, corresponding to previously described intraepithelial ILC1s (32). The recent identification of the NKp44 receptor, moreover, indicates an important role of Nkp44 in viral infections (33). NKp30 is another interesting NCR in viral infections due to its capacity to interact with dendritic cells (DCs) and impact on the NK-DC cross-talk (34–36).

There is an urgent need for a better characterization of these NK cell subpopulations in lymphoid tissues. Until now, it has been very difficult to relate our knowledge of human NK cell biology to that of NHP and translate this information into pre-clinical practice. Among the different NHP models, African NHPs, such as AGM, have been a natural host for simian immunodeficiency viruses (SIVs) for probably more than one million of years (37–39). In contrast to people living with HIV (PLH), natural hosts for SIV typically do not progress toward disease despite persistent high viremia (40). At the opposite, Asian macaques are not natural hosts of SIV, and experimental infection with SIVmac induces AIDS preceded by rapid loss of CD4^+^ T cells in intestinal mucosa and chronic systemic inflammation as in PLH. We have previously shown that in contrast to HIV-1 infected humans and SIVmac-infected macaques, AGMs infected with SIVagm are free of chronic inflammation and efficiently control viral replication in secondary lymphatic tissue (SLT) (41). The viral control in SLT of SIVagm-infected AGMs is mediated predominantly by NK cells in a CXCR5-dependent manner (41). Paradoxically, no viral control is observed in the intestine of SIVagm-infected monkeys (42). AGMs, therefore, represent a model of tissue-specific NK cell-mediated viral control.

In this study, we comprehensively determined tissue-specific frequencies, phenotypes, and potential activity profiles of NK cells in major NHP models. We investigated NK cells in blood, liver, lymph nodes, spleen, ileum, jejunum, colon, and rectum from uninfected animals and after infection with SIV. This work provides clues to understand better the behavior of tissue NK cells and delivers novel tools for the study of therapeutic and vaccinal approaches in NHP models for human diseases.

## Materials and Methods

### Monkeys and Simian Immunodeficiency Virus Infections

Samples from a total of 36 NHPs were used in this study distributed across four different species. The number of animals per species was as follows: 13 AGMs (Caribbean *Chlorocebus sabaeus*), 12 CMs (*Macaca fascicularis*), 7 RMs (*Macaca mulatta*), and 4 baboons (*Papio anubis*). Seven CMs and seven AGMs were infected with SIVmac251 and SIVagm.sab92018, respectively, as previously described (23, 41). The viremia levels are shown in Supplementary Figure S2. All infected monkeys were highly viremic. All the animals were free of simian retrovirus type D and simian T-lymphotropic virus type 1.

The AGMs, CMs, and baboons were housed at the IDMIT Center (Fontenay-aux-Roses, France), and the RMs at the German Primate Center (DPZ). All experimental procedures were conducted in strict accordance with the International European Guidelines 2010/63/UE on the protection of animals used for experimentation and other scientific purposes and with the national laws (French Decree 2013-118). The IDMIT Center complies with the Standards for Human Care and Use of the Office for Laboratory Animal Welfare (United States) under Office for Laboratory Animal Welfare Assurance number A5826-86. Monitoring of the monkeys at IDMIT was under the supervision of the veterinarians in charge of the animal facilities. The Ethical Committee of Animal Experimentation (CETEA-DSV, IDF, France) (notification 12-098 and A17-044) approved experimental animal protocols. The DPZ is allowed to breed and house NHPs under license number 392001/7 approved by the local veterinary office and conforming with § 11 of the German Animal Welfare act. The Lower Saxony State Office for Consumer Protection and Food Safety with the project license 33.9-42502-05-10A041 granted the collection of blood samples at the DPZ. In compliance with the principles of the three Rs (replacing, reducing, and refining), all samples used here were from animals that were shared with other studies. Indeed, the animals have been purchased or infected for other programs. For instance, we used tissues that were collected from animals that have been killed for other studies, and some of these have already been published (4, 41, 81).

Monkeys were sedated with ketamine chlorhydrate (Rhone-Mérieux, Lyons, France) before handling. Sample collection was performed in random order, according to the tripartite harmonized International Council for Harmonization of Technical Requirements for Pharmaceuticals for Human Use (ICH) Guideline on Methodology (previously coded Q2B). The investigators were not blinded, whereas the animal handlers were blinded to group allocation.

### Tissue Collections and Processing

Whole venous blood was collected in ethylenediaminetetraacetic acid tubes. Ficoll density-gradient centrifugation isolated peripheral blood mononuclear cells. Biopsies of pLNs were performed by excision. Other tissues were collected at autopsy. After careful removal of adhering connective and fat tissues, pLNs and spleen cells were dissociated using the gentlemacS^TM^ Dissociator technology (Miltenyi Biotec, Germany). The cell suspension was subsequently filtered through 100- and 40-μm cell strainers, and cells were washed with cold phosphate-buffered saline. Cells were either immediately stained for flow cytometry or cryopreserved in 90% fetal bovine serum and 10% dimethyl sulfoxide and stored in liquid nitrogen vapor.

### Viral RNA Quantification

The viral RNA copy numbers in plasma of the animals were quantified as previously described (23, 41).

### Polychromatic Flow Cytometry

For flow cytometric analysis, cells were stained in U-bottom tubes in the dark. Cells were analyzed using 11-parameter flow cytometry. For surface staining, cells were incubated in phosphate-buffered saline with the appropriate monoclonal antibodies and Zombie Aqua^TM^ for 30 min at 4°C, washed and fixed with 4% paraformaldehyde. The following monoclonal antibodies were used for surface and intracellular staining (clone, suppliers): CD3 (SP34-2, *BD Biosciences*), CD8 (BW135/80; Miltenyi Biotec), CD20 (2h7; eBioscience), CD16 (3G8,Coulter), NKG2A (Z199, Coulter), NKP30 (AF29-4D12, Miltenyi Biotec), CD45 (D058-1283, *BD Biosciences*), CD69 (FN50, *BD Biosciences*), NKP46 (BAB281,Coulter), CXCR5 (MU5UBEE, Thermo), CD279 (PD-1) (EH12.2H7, Biolegend), CXCR3 (1C6/CXCR3, *BD Biosciences*), IFN-γ (4S.B3,*BD Biosciences*), GzmB (GB11, *BD Biosciences*), TNF-A (MAB11, *BD Biosciences*), NKP44 (REA1163, Miltenyi Biotec), and CD14 (TÜK4, Miltenyi Biotec). Reference listing the cross-reactivity of each antibody is listed in Supplementary Table S1. The anti-NKG2A antibody used recognizes both NKG2A and NKG2C on simian cells. Due to the lack of information on the KIR polymorphism in African NHP and the rare anti-KIR antibodies specific for NHP, the KIR expression has not been analyzed. Flow cytometry acquisitions were performed on an LSRII (BD Biosciences). The data were further analyzed using FlowJo 10.4.2 software (FlowJo, LLC, Ashland, OR, United States). Intracellular staining was performed using BD Cytofix/Cytoperm^TM^. Uniform manifold approximation and projection was performed with Flojow, using 1,000 iterations and perplexity of 0.25.

### Statistics

**FIGURE 1:**
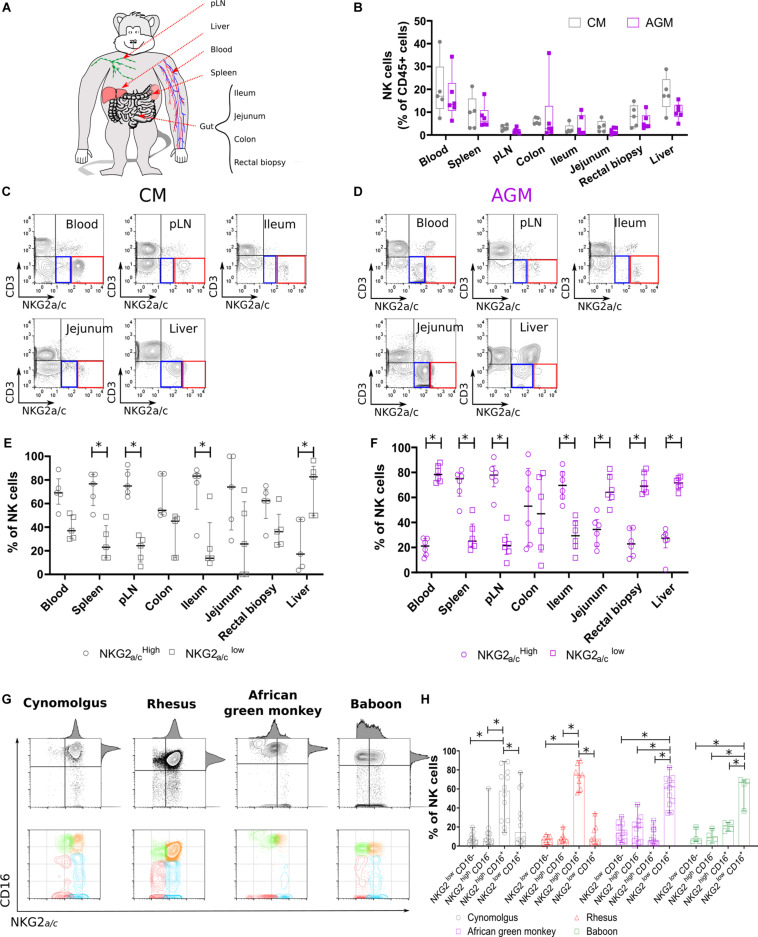
Non-human primate NK cell subset distributions in tissues. **(A)** Scheme of the NHP tissues obtained and experimental workflow presented in this study. **(B)** NK cell distribution in distinct tissue compartments. Frequencies are shown in boxplots and correspond to compiled data from 5 SIV negative CMs (gray) and 6 SIV negative AGMs (purple). **(C,D)** Representative flow cytometry plots for NKG2a/c^LOW^ (blue square) and NKG2a/c^HIGH^ (red square) NK cell subsets in multiple tissue sites obtained from **(C)** CM and **(D)** AGM. **(E,F)** Distribution of NKG2a/c^LOW^ (empty circle) and NKG2a/c^HIGH^ (empty square) NK cell subsets in multiple tissue sites obtained from **(E)** five CMs and **(F)** six AGMs. **(G)** Representative dot plots showing blood NK cell subset distributions according to NKG2a/c and CD16 in four NHP species as indicated. **(H)** Frequency of blood NK cell subsets in four SIV negative NHP species. Each symbol depicts an individual animal among the 13 AGM, 12 CM, 7 RM, and 4 baboons. Median and standard error bars are shown. Statistical significance of differences was assessed using non-parametric Mann–Whitney *U* tests **(B)**, or Wilcoxon matched-pairs signed-rank test, for paired samples **(E,F,H)**. Asterisks indicate a *p*-value <0.05.

**FIGURE 2:**
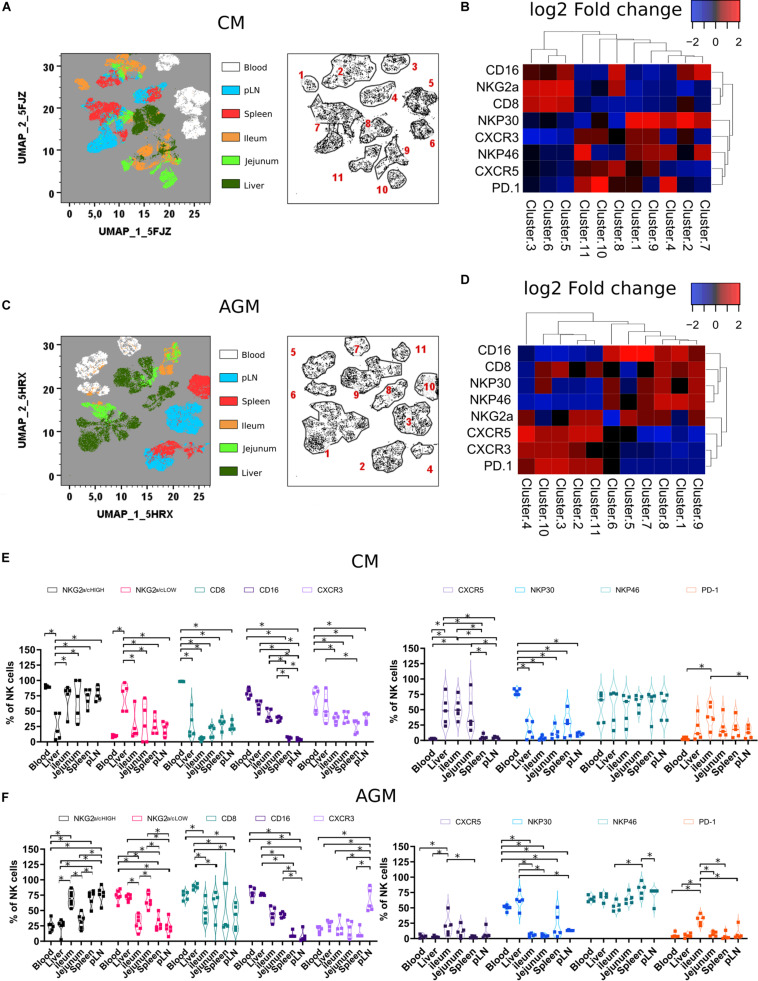
Tissue localization shapes the phenotype of NK cells **(A)** Force-directed plot showing the relationship between NK cells from distinct tissues identified by multidimensional analysis (data compiled from five healthy CM). Each node is an aggregation of phenotypically similar cells. Resulting from several steps, including downsampling, merging, and overall dimension reduction followed by gating (left panel), and collection of similar nodes forms 11 distinct NK cell clusters (right panel). **(B)** Heatmap showing the differential expression of cell surface markers used for clustering and community identification in the multidimensional analysis in panel **(A)**. **(C)** Same analyses as in panel **(A)** performed on six healthy AGM. **(D)** Heatmap showing the differential expression of cell surface markers used for clustering and community identification in the multidimensional analysis in panel **(C)**. **(E,F)** Vioplots showing the distribution of markers on bulk tissue NK cells from **(E)** healthy CMs and **(F)** healthy AGMs. Each marker is labeled with a unique color. All comparisons were carried out using a one-way analysis of variance (ANOVA) and followed by a Tukey’s multiple-comparison test. The latter is a *post hoc* test based on the studentized range distribution. Median and standard error bars are shown. Asterisks indicate a *p*-value <0.05.

**FIGURE 3:**
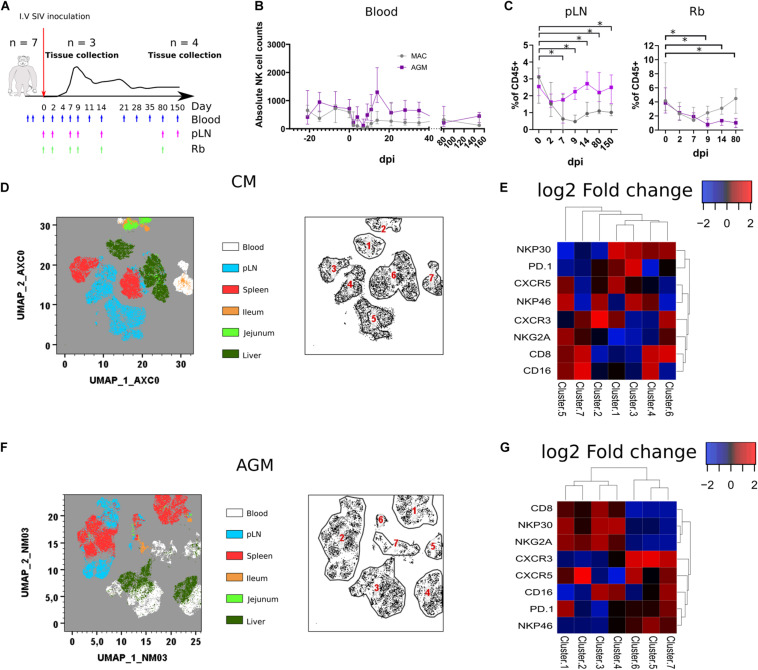
Rapid change in tissues NK cell phenotypes following SIV infection. **(A)** Schematic representation of the SIV infection schedule used in this study. Seven CMs and seven AGMs were intravenously injected with SIV (see section *Method*). Blue arrows indicate blood sampling. Purple and green arrows indicate pLN and rectal biopsy (Rb) samplings, respectively. Three monkeys per species were euthanized at day 9 p.i., and the remaining animals were euthanized during the chronic phase (day 240 p.i.). **(B)** Follow-up of blood NK cell absolute counts before and during SIV infection in CM (gray line) and AGM (purple line). **(C)** Frequency of NK cells among CD45 + cells in pLN (left panel) and Rb (right panel) during SIV infection in CM (gray line) and AGM (purple line). **(D)** Force-directed plot showing the relationship between NK cells from distinct tissues at day 9 p.i. identified by multidimensional analysis (data compiled from three CMs). Each node is an aggregation of similar cells resulting from the downsampling of data (left panel), and the collection of similar nodes forms six distinct NK cell clusters (right panel). **(E)** Heatmap showing the differential expression of cell surface markers used for clustering and community identification in the multidimensional analysis in panel **(D)**. **(F)** Same analyses as in panel **(A)** performed on three AGMs at day 9 p.i. **(G)** Heatmap showing the differential expression of cell surface markers used for clustering and community identification in the multidimensional analysis in panel **(F)**. Median and standard error bars are shown. Asterisks indicate a *p*-value <0.05.

**FIGURE 4:**
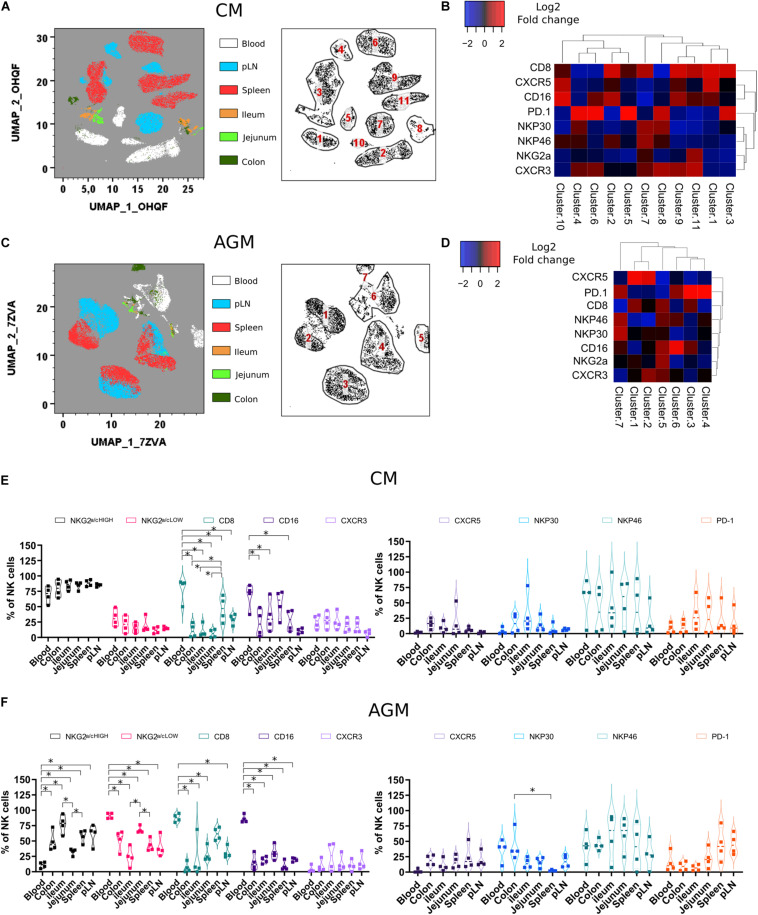
Phenotypic analysis of NK cells during chronic SIV infection across different tissue sites. **(A)** Force-directed plot showing the relationship between NK cells from distinct tissues identified by multidimensional analysis (data compiled from five chronically infected CMs). Each node is an aggregation of similar cells resulting from the downsampling of data (left panel), and the collection of similar nodes forms 11 distinct NK cell clusters (right panel). **(B)** Heatmap showing the differential expression of cell surface markers used for clustering and community identification in the multidimensional analysis in panel **(A)**. **(C)** Same analyses as in panel **(A)** performed on four chronically infected AGMs. **(D)** Heatmap showing the differential expression of cell surface markers used for clustering and community identification in the multidimensional analysis in panel **(C)**. **(E,F)** Vioplots showing the distribution of markers on bulk tissue NK cells from four chronically SIV-infected CMs **(E)** and four chronically SIV-infected AGMs **(F)**. Each marker is presented with a unique color. All comparisons between NK cells were carried out using a one-way analysis of variance (ANOVA) and followed by a Tukey’s multiple-comparison test. The latter is a *post hoc* test based on the studentized range distribution. Median and standard error bars are shown. Asterisks indicate a *p*-value <0.05.

**FIGURE 5:**
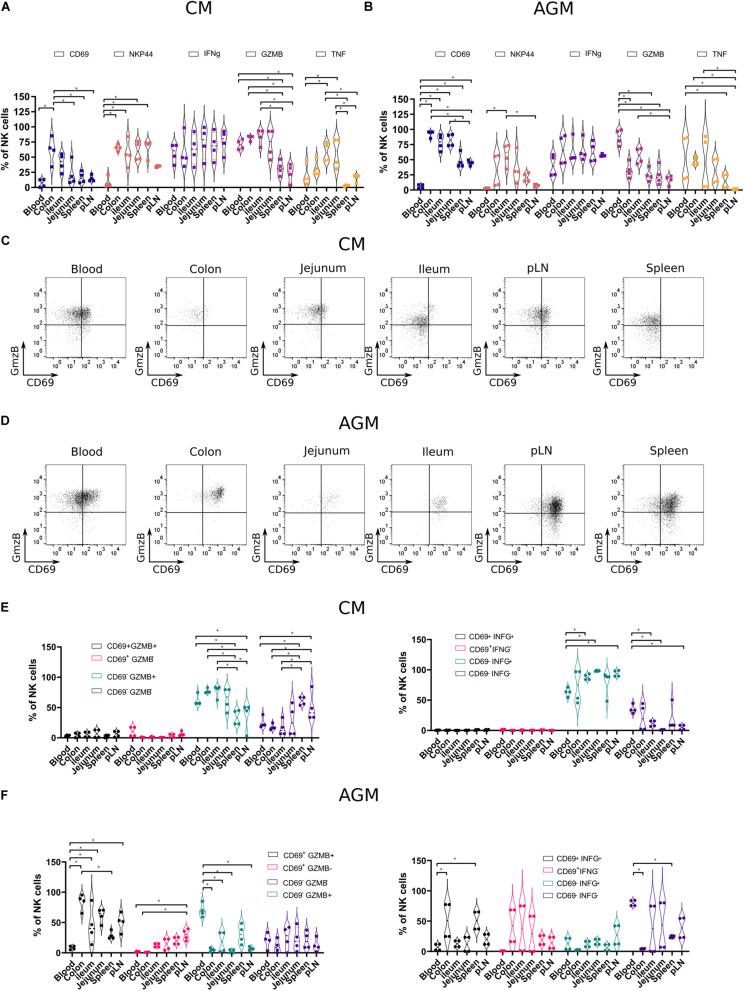
Functional profiles of NK cells in tissues from chronically SIV-infected non-human primates. NK cells from four SIVmac-infected CMs and four SIVagm-infected AGMs were analyzed. **(A,B)** Vioplots showing the distribution of markers on bulk tissue NK cells. Each marker is labeled with a unique color. All comparisons between NK cells were carried out using a one-way analysis of variance (ANOVA) and followed by a Tukey’s multiple-comparison test. The latter is a *post hoc* test based on the studentized range distribution. **(C,D)** Representative dot plots showing NK cells according to CD69 surface expression and intracellular expression of GzmB in different tissues. Dot plots for a randomly chosen animal per species are shown. **(E,F)** Graphics showing frequencies of NK cells subsets in different tissues according to CD69 and Grzm B expression (left) or CD69 and IFN-g expression (right). All comparisons were carried out using a one-way ANOVA and followed by a Tukey’s multiple-comparison test. The latter is a *post hoc* test based on the studentized range distribution. Median and standard error bars are shown. Asterisks indicate a *p*-value <0.05.

The GraphPad Prism 7 (GraphPad Software, San Diego, CA) was used to analyze data and to perform statistical analyses. Statistical significance of differences was assessed using non-parametric Mann–Whitney *U* tests (Figures 1A, 3B,C) or Wilcoxon matched-pairs signed-rank test for paired samples (Figures 1E,F,H). All group comparisons were carried out using a one-way analysis of variance and followed by a Tukey’s multiple-comparison test. The latter is a *post hoc* test based on the studentized range distribution (Figures 2E,F, 4E,F, 5A,B,E,F). Values of *p* < 0.05 were considered significant. NK cell populations were analyzed by the uniform manifold approximation and projection method provided by the FlowJo plugin version 3.1. Heatmap of the Log2(FC) of the marker expression was compared with the median across all samples. It was color-coded with blue for lower expression and red for higher expression. Dendrograms present the clustering of samples (columns) and markers (row), which is based on hierarchical clustering with Euclidean distance metric and average linkage.

## Results

### Tissue-Intrinsic Distribution of Non-human Primate Natural Killer Cell Subsets

To analyze NK cell distribution and phenotype profiles across different anatomic sites in Asian and African NHPs, we collected blood, secondary lymphoid organs (spleen and peripheral lymph nodes [pLNs]), liver, and gut (small and large intestines) from cynomolgus macaques (CMs) and AGMs (Figure 1A). NK cells were defined as previously reported by us and others (20–22, 41) as CD45^+^CD3^–^CD14^–^CD20^–^NKG2_a/c_^+^ cells. The tissue distribution was analyzed first in healthy CM and AGM. The distribution was consistent between animals for a given species with blood and liver having significantly higher frequencies of NK cells (5–40%) compared with low frequencies (<0.1–5%) of NK cells in the pLNs and gut (Figure 1B).

NK cells were further delineated into two NK cell subsets, NKG2a/c^HIGH^ and NKG2a/c^LOW^ (Figures 1C,D and Supplementary Figure S1). The distribution of NKG2a/c^HIGH^ and NKG2a/c^LOW^ NK cell subsets was also dependent on the tissue site (Figures 1C,D). NKG2a/c^HIGH^ NK cells predominated in pLNs, spleen, and ileum, whereas NKG2a/c^LOW^ NK cells comprised the majority of NK cells in liver independently of the species (Figures 1E,F). Thus, NKG2a/c^HIGH^ NK cells were more prevalent in sites known for containing an abundance of immature NK cells such as pLN, spleen, and gut, and in the liver, potentially mature NK cells were predominant.

We also observed a difference between the species, as NKG2a/c^HIGH^ NK cells were the main population in blood, jejunum, and rectum of CM in contrast to AGM where NKG2a/c^LOW^ NK cells dominated in these three compartments (Figures 1E,F). To clarify the difference observed between these two species, we analyzed a higher number of animals and more species (Figure 1G). Thus, blood NK cells were analyzed in 12 CMs, 11 AGMs, 8 rhesus macaques (RMs), and 4 baboons. The NKG2a/c^HIGH^ NK cells were again the major population in the blood of the two Asian NHPs (CM and RM) and not in the two African NHPs (AGM and baboon) (Figure 1H).

To summarize, the NK cell distributions across tissues varied, similar to the ones described in humans (5). The frequencies of total NK cells in different anatomical sites did not differ between NHP species. The frequency of NKG2a/c^LOW^ NK cells in blood was higher in the NHP species from Africa compared with that in the macaque species.

### Evaluation of Natural Killer Cell Diversity in Tissues From Non-human Primates

To further analyze the difference between the tissues, we implemented a high-dimensional flow cytometry analysis of simian NK cells, allowing notably the measurement of NCRs, homing receptors, and cellular exhaustion marker (PD-1). We included, as homing receptors, CXCR3 and CXCR5, which are, respectively, a major tissue homing receptor for NK cells (43) and a B cell follicle homing receptor for lymphocytes (44–46). The NK cells from six anatomical sites (blood, pLN, spleen, liver, ileum, and jejunum) collected from five healthy CMs and four healthy AGMs were analyzed. A heatmap based on the mean fluorescence intensity for each marker at the cluster level was generated. Force-directed clustering analysis resulted in 11 distinct NK cell clusters for both AGM and CM (Figures 2A–D). In both species, blood NK cells segregated into three distinct subclusters that did not reveal overlaps with NK cells from tissues. Either the liver NK cells formed only one cluster or the clusters grouped together, generally separate from other tissues. Spleen and pLN NK shared some clusters. The jejunum and ileum NK cells not only appeared to be closely linked but also sometimes were found in clusters with other tissue NK cells (Figures 2A–D).

When comparing the two species, NK cells from pLN and spleen in AGM showed elevated levels of CXCR5 expression compared with other tissues. In contrast, for CM, the highest CXCR5 expression was observed in the gut (Figures 2B,D). CM also showed high frequencies of CXCR5 + NK cells in the liver (Figures 2E,F). Another distinct feature of the CM was a high frequency of NK cells with lower CD8 expression in tissues (Figure 2E).

In summary, NK cells showed tissue-specific clusters with LN and spleen NK cells closely related. Tissue NK cells in CM rarely expressed CD8. In the intestine and liver, NK cells in CM frequently expressed CXCR5.

### Tissue Natural Killer Cells Are Differentially Impacted in Pathogenic Compared With Non-pathogenic Simian Immunodeficiency Virus Infection

We evaluated if and how SIV infection modulates NK cell distribution and phenotype among tissues. We included in the analysis of tissue homing markers and compared the distribution of NK cell subpopulations between the tissues. Seven CMs and seven AGMs were followed during SIVmac and SIVagm infection, respectively (Supplementary Figure S2). NK cells from blood, pLN, and rectal biopsy were longitudinally monitored during the time course of infection (Figure 3A). To have access also to liver, ileum, and jejunum during acute infection, we analyzed tissues collected at necropsy from three monkeys of each species at day 9 post-infection (p.i.) and at the time of peak viremia and from four animals of each species during the chronic phase of infection (day 240 p.i.). Assessment of absolute NK cell counts in blood did not reveal statistically significant differences during SIV infection, even if a trend for the decrease was observed in each species during an acute infection—up to day 3–4 p.i. (Figure 3B). In pLN, the frequency of NK cells rapidly decreased in CM during acute infection to never reach the level observed before infection throughout chronic infection, whereas no statistically significant change was observed in AGM (Figure 3C, left graph). In contrast, AGM NK cells in rectal biopsies showed a constant decrease during infection, whereas CM NK cells tended to drop at day 7 p.i. but only transiently and remained normal after that (Figure 3C, right graph).

Force-directed clustering analysis of NK cells from six tissues obtained at day 9 p.i. generated seven distinct NK cell clusters per species (Figures 3D–G). NK cells clustered again according to tissues as before infection, except some overlap between liver and blood or pLN. The highest density of expression was observed for CXCR5 on NK cells from SLT in AGM, whereas it was also elevated in some liver NK cells in CM. CXCR3 expression was high on NK cells from blood and SLT in AGM, whereas high on intestinal and blood NK cells for CM, suggesting that CXCR3 might be involved in the distinct tissue distribution of NK cells after SIVmac and SIVagm infection.

We then performed the same analysis on chronically infected animals. Due to the decrease and few numbers of NK cells in gut tissues from AGM during SIVagm infection (Figure 3C, right graph), we included an additional intestinal compartment (the colon) at the expense of the liver in the analyses (Figures 4A–E). Whereas, in healthy animals, CXCR3 and CXCR5 were found in the same clusters (Figures 2B,D), they clustered separately in infected animals (Figures 4A–D), indicating that the CXCR3 + and CXCR5 + NK cells evolved differentially after SIV infection. In SIVmac infection, the frequencies of CXCR5 + NK cells in the gut decreased (Figure 4E) as compared with those before infection (Figure 2E). CXCR5 expression on NK cells was also low in SLT of CM compared with AGM in chronic SIV infection (Figures 4A–D), correlating with previous data on the higher levels CXCR5 + NK cells in SLT during chronic SIVagm infection as compared with chronic SIVmac infection (41).

PD-1 is known to be expressed on exhausted CD8 + T cell lymphocytes. However, PD-1 expression is also high on CXCR5 + lymphocytes residing in the B cell follicles of SLT (T_FH_ and B cells). On NK cells, as shown here, PD-1 was often expressed on the same cell subset of CXCR5 + NK cells before infection but mostly on distinct NK cell subsets after SIV infection (Figures 4B,D). PD-1 + NK cells were increased in SLT during SIVagm infection (Figure 4F) as compared with healthy animals (Figure 2F), which might be related to their accumulation in B cell follicles during chronic SIVagm infection (41).

All over, we demonstrate a distinct tissue distribution of NK cells after SIV infection as compared with healthy animals. Also, the dynamic of NK cell frequencies were opposite between AGM and CM: decrease in SLT for CM and decrease in the gut for AGM after SIV infection.

### Resident Tissue Natural Killer Cells Express a Cytotoxic Phenotype in the Natural Host of Simian Immunodeficiency Virus Infection

Next, we analyzed the phenotype of the NK cells in distinct tissues. We performed the first analysis on total NK cells and then on NK cells that express markers that have been associated with tissue residency of T cells, such as CD69, and those present on innate mucosal lymphocytes, such as NKp44. We assessed the distribution of CD69 + and NKP44 + NK cells in distinct tissues from SIV-infected animals (Figures 5A,B). CD69 + NK cells were generally more frequent in tissues than in blood, in particular in the colon of CM (34 to 85%) (Figure 5A) and in all analyzed tissues in AGM (pLN, spleen, ileum, jejunum, and colon) (Figure 5B). As for CM, the highest frequencies of CD69 + NK cells in AGM were observed in the intestine compared with secondary lymphoid organs and blood (Figure 5B). NKP44 + NK cells were rare in blood and LN, whereas frequent in the intestine (Figures 5A,B). CM also harbored NKp44 + NK cells in the spleen. Altogether, CD69 + and NKp44 + NK cells were generally observed more often in tissues, and Nkp44 + NK cells were more frequent in the gut than in SLT, in particular in AGM, suggesting that these markers can be used in a similar way as in humans.

We evaluated the cytotoxic phenotype of the NK cells between the distinct tissues and combined the analysis with effector molecules: granzyme B (GzmB), interferon-gamma (IFN-g), and tumor necrosis factor-alpha (TNF-a). We performed *ex vivo* intracellular staining for GzmB, IFN-g, and TNF-a in the healthy AGMs and CMs (Figures 5A,B). We thus avoided any potential bias of *in vitro* cultures and provided profiles the closest to the physiological patterns *in vivo*. We observed high levels of IFN-g + NK cells in all compartments (Figures 5A,B). In contrast, the frequencies of TNF-a + NK cells and GzmB + NK cells varied according to the tissues. The frequencies of GzmB + NK cells were high in blood from both CMs (65–80%) and AGMs (72–96%) and low in SLT (Figures 5A,B). In the intestine, GzmB + NK cells were sometimes as frequent as in blood, in particular in CM, and sometimes at intermediate levels between SLT and blood. Similar to GzmB + NK cells, TNF-a + NK cells were rare in SLT and sometimes frequent in the gut. There was, however, a high interindividual variability, for example, for TNF-a + NK cells in the blood. Altogether, NK cells with a cytotoxic phenotype were seen more often in effector sites (blood and gut) than in inductive sites (pLN and spleen). IFN-g + NK cells were observed in all compartments, including in inductive sites (SLT).

Finally, we studied if there was a link between tissue-specific residency of NK cells and their effector profiles. We analyzed, therefore, the *ex vivo* expression of IFN-g and GzmB according to the expression of the potential tissue residency marker CD69 in the distinct lymphoid compartments of both species (Figures 5C,D). In SIV-infected CMs, most of the GzmB + cells did not express CD69, irrespective of the tissue studied (Figure 5E). These CD69^–^GzmB + NK cells in CM were more frequent in the intestine (colon and ileum) than in SLT. In AGM, the GzmB + NK cells in blood were CD69- but in tissues CD69 + (Figure 5F). Similar to GzmB, the IFN-g + NK cells in SIV-infected CM were mostly CD69-, whereas in the SIV-infected AGM, many IFN-g + NK cells were CD69 +. In the SIV-infected CM, most NK cells were IFN-g + in all lymphoid tissues analyzed, in contrast to the SIV-infected AGM, where most NK cells were IFN- g-, in particular in the intestine. Altogether, most GzmB + and IFN-g + NK cells in the SLT and intestine of SIV-infected CMs were CD69-, whereas CD69 + in AGMs. Given that NK cells were increased in the intestine of CM in chronic SIV infection as compared with AGM (Figure 3C, right panel), this could suggest that most of the NK cells in the intestine during chronic SIVmac infection were infiltrating and not resident NK cells.

## Discussion

In this study, we set out to investigate the tissue distribution, phenotype, and function of NK cells in tissues of healthy and SIV-infected NHPs. Moreover, we analyzed tissue-residence markers combined with effector molecules inside tissues. This study allowed to identify tissue- and species-specific patterns of NK cell frequencies and phenotype in lymphoid and non-lymphoid tissues of primates.

Studies on NK cells in lymphoid tissues are scarce because NK cell frequencies are low in these tissues. However, it is important to gather more information about NK cells in such tissues, as their numbers and functions can be modulated by viral infections and can also be given the importance of the NK-DC cross-talk in LN for shaping the adaptive immune responses (47, 48). Although we did not analyze all possible tissues, we focused the study on lymphoid tissues where there is little information and that are key for the study of vaccine responses, i.e., mucosa as a site of viral entry and replication and SLT for the education of B and T cell responses. We compared here the NK cells present in such anatomic sites with body compartments where NK cells are more frequent and more often described (blood and liver).

We performed these analyses in representative species from Asia and Africa. We, thus, studied four NHP species and only two of them extensively: one Asian (CM) and one African (AGM) species. Of note, these two species are among the most used NHP models in biomedical research. Also, the data observed here for CM might be similar in related species, such as RMs.

Here, we performed a multiparameter flow cytometry. Even if we did not use next-generation sequencing technology, such analysis allowed us to focus on specific markers and to analyze a wide range of tissues (blood, liver, spleen, lymph nodes, ileum, jejunum, colon, and rectum) concomitantly. We also performed a longitudinal analysis in two distinct tissues (SLT and intestine) in response to a viral infection. Most importantly, we provide *ex vivo* data for NK cell functionality in these tissues.

Our data demonstrate that NK cells of CM were rarely CD8 + in tissues. Previous reports have shown that CD8 engagement on NK cells delivers an activating signal that increases the synthesis and secretion of IFN-g (49). CD8 may deliver an activating signal only when it is expressed at a high density (50). KIR and/or C-type lectin inhibitory receptors such as NKG2a/c can downregulate CD8-mediated triggering (49). The downregulation of CD8-dependent IFN-g production exerted by inhibitory receptor superfamily members may represent a mechanism to limit NK cell responses, as it has been proposed for the inhibiting signal delivered through inhibitory receptors to avoid lysis of autologous cells expressing the appropriate human leukocyte antigen (HLA)-I ligand of a given inhibitory receptor superfamily member (51). Other NK cell surface receptors, including NKp30 and NKp44 (52–54), could thus rather be involved in recognition of target cells and activation of NK cell-mediated lysis in CM tissues. Our data also imply that studies using anti-CD8a antibodies to deplete NK cells need to consider the low frequency of CD8 + NK cells in tissues of some species. Indeed, our study indicates that in CM, NK cells will not get efficiently depleted in tissues when using anti-CD8 antibodies.

CXCR5 is a chemokine receptor mainly expressed on B cells, as well as DCs and T cell subsets such as T_FH_ cells (55, 56) present in B cell follicles. We have recently provided evidence that the capacity to control SIV viral replication in B cell follicles of AGM was associated with the presence of CXCR5^+^ NK cells (41), showing that CXCR5 could be also be frequently expressed on NK cells under some circumstances. The role of CXCR5 in the gut is less studied (57). Nevertheless, in the gut, CXCR5 is known to support solitary intestinal lymphoid tissue (SILT) formation and B cell homing (58). Here, we show that CXCR5 is expressed to high frequency by ileum NK cells in healthy CM and AGM. This high frequency of CXCR5 + NK cells was also noticed in CM jejunum and colon. Strikingly, after SIV infection, the frequency of CXCR5 + NK cells strongly decreased in the gut of CM for unknown reasons. It could be related to the increase of infiltrating, CXCR5- NK cells. Microbiota and other external stimuli can foster the formation of aberrant SILT distinguished by impaired development of B cell follicles in CXCR5-deficient mice (58–60). In this context, it could be interesting to investigate if the decrease of CXCR5 + NK cells in the intestine during SIVmac infection, concomitant with the well-known induction of microbial translocation, may be associated with the formation of SILT in NHPs and humans during HIV and SIVmac infections.

Although CD69 has long been viewed as an activation marker of both T and NK cells, it is now clear that it has an important role in retaining immune cells in tissues by inhibiting sphingosine-1-phosphate receptor 1 (61, 62). CD69 is now considered a tissue-resident marker for T cells (63, 64). For NK cells, it is less clear if tissue-resident NK cells can be characterized based on the expression of CD69. It has recently been shown that the CD69 expression displays subset- and site-specific variations by tissue NK cells in humans (5). Here, we show that during SIV infection in NHP, NK cells in peripheral blood largely lack expression of CD69. Our data in the tissues argue in favor of CD69 as a tissue residence marker for NK cells in NHP. The main difference between the pathogenic and non-pathogenic SIV infections in the two species (CM and AGM) came from the observation that the frequency of CD69 cells in tissues during pathogenic infection was relatively low, whereas in the natural host, a high frequency of CD69 + NK cells was observed in tissues. The high frequency of CD69- NK cells in SIVmac infection might be the result of infiltrating NK cells. The combined analysis of CD69 with GzmB + and IFN-g indicates that most of resident NK cells harbored a cytotoxic profile, whereas potentially infiltrating (CD69-) NK cells also expressed the pro-inflammatory IFN-g cytokine. It is not clear though if these changes are the result of blood NK cell infiltration or whether there are phenotypic changes in resident NK cell populations during SIV infection.

The role of tissue localization in NK cell development and function and how circulating NK cells relate to those in different sites are not well understood during HIV/SIV infection (22, 65, 66). Only a few data on NK cells in tissues are available during HIV/SIV infections (41, 65, 67). Immediately after HIV/SIV infection, changes occur in NK cell populations in the gut-associated lymphoid tissue. Thus, the frequency of both intraepithelial and lamina propria NK cells was increased in PLH with an incomplete blood CD4 + T cell recovery (CD4 < 350 cells/μl) despite several years of effective antiretroviral treatment (68). Similarly, an increase in total and NKG2a/c + NK cells in rectum RM has been shown during SIVmac infection (66, 69). In contrast, the NKp44 + lymphocytes were significantly depleted in acute HIV and SIV infections in the mucosa and continued to decline in frequency during chronic phase (70). Antiretroviral therapy significantly increased the frequency of mucosal NKG2a/c + NK cells (71). SIV infection in pathogenic models drives trafficking away from secondary lymphoid organs toward the intestine (22, 72, 73). This is related to the upregulation of the gut-homing marker α_4_β_7_ on NK cells, as shown in chronic SIVmac infection coupled with downregulation of the LN-trafficking marker CCR7 (66, 74). Our data in the CM are in line with these reports. Strikingly, we observed opposite tissue dynamics in heterologous and natural hosts of SIV. In the natural host, NK cells were not directed toward the intestine, and the frequencies of total NK cells in the gut were decreased in SIVagm infection in contrast to SIVmac infection. This could explain the lack of NK cell-mediated viral control in the intestine of SIVagm-infected AGMs. In CM, NK cells decreased in the LN but increased in the gut in contrast to SIVagm infection in AGM. These distinct NK cell tissue distributions were associated with CXCR3 expression, supporting its role as an inflammatory tissue homing marker for NK cells. The higher levels of the CXCR3 ligand (IP-10) in the intestine of the SIVmac-infected macaques as compared with those of the SIVagm-infected AGMs (75, 76) might explain in part, together with previously reported upregulations of other receptors such as α_4_β_7_, the higher levels of NK cells in the intestine of the SIVmac-infected macaques as compared with the natural host. The fact that NK cells seem to be redirected to the intestine in SIVmac infection raises the question of their inefficient activity against the cells replicating the virus in the gut. In fact, an increase in these cells in this compartment should favor a better control of the viral replication. However, beyond the mechanisms established by the virus to escape NK cell lysis, the inflammatory environment, as well as the loss of regulatory cells (i.e., ILCs, TH17, CD4 T cells), might profoundly and durably alter NK cell functions. It has also previously been reported that NKG2a/c + NK cells accumulate in vaginal mucosa during acute SIVmac infection and that this occurred concomitant with an increase in HLA-E that could induce an inhibitory signal to the NKG2a/c + NK cells (77). Alternatively, it is not excluded that NK cells directed to the gut contribute to tissue damage in the intestine (78). It has indeed been demonstrated in the macaque that microbial translocation increased the recruitment of cytotoxic NK cells in the liver (78). This increase in cytotoxic NK cells resulted in accelerated destruction of this organ. Recently, NKp44 + NK cells have been implicated in autoimmune diseases, such as inflammatory bowel disease or Crohn disease (79); however, whether they participate in pathologic or protective processes of chronic inflammation *in vivo* remains controversial. HLA-DP molecules have recently been shown to bind NKp4433 directly. HLA-DP is constitutively expressed on antigen-presenting cells, including B cells. HLA-DP molecules are upregulated on non-hematopoietic tissues in response to inflammation (80). Thus, it might be relevant to investigate the impact of NKP44 + NK cells on gut homeostasis in the context of gut inflammation, such as HIV infection. The potential contribution to tissue damage and homeostasis by NK cells during chronic HIV/SIVmac infections will need to be addressed in future studies.

Given the remaining necessity in the use of NHP in biomedical research, as shown by the recent severe acute respiratory syndrome coronavirus 2 outbreak, these data are highly relevant for establishing the validity and utility of such models for studying the role of NK cells in human diseases. More and more studies raise the question of the utility of NK cells in antitumor and antiviral immunotherapies. Our data on the NK cell tissue distribution and functional profiles in the NHP contribute to the understanding of their role in lymphoid tissues during HIV infection as well as to efforts in the development of vaccines and therapies against human diseases.

## Data Availability Statement

All datasets presented in this study are included in the article/Supplementary Material.

## Ethics Statement

The animal study was reviewed and approved by the Ethical Committee of Animal Experimentation (CETEA-DSV, IDF, France) (Notification 12-098 and A17-044 and license number 392001/7).

## Author Contributions

NH and MM-T designed the study. NH designed the experiments. NH, PR, CP, and BJ performed the experiments. CS-H and A-SB provided the samples. NH performed the statistical analyses. NH, PR, CP, J-LP, A-SB, BJ, and MM-T analyzed the data. VC, RL, and BJ coordinated the animal studies. MM-T obtained the funding. NH and MM-T wrote the manuscript. All co-authors reviewed the manuscript. All authors contributed to the article and approved the submitted version.

## Conflict of Interest

The authors declare that the research was conducted in the absence of any commercial or financial relationships that could be construed as a potential conflict of interest.

## Supplementary Material

FIGURE S1 Gating strategy used to define NK cell subsets in the NHP.

FIGURE S2 Viremia profiles during SIV infections. Seven CM and seven AGM were respectively infected with SIVmac251 and SIVagm.sab92018. The SIV copy numbers in the plasma over time are indicated.
