# Supplementary material for: Non-human Primate Determinants of Natural Killer Cells in Tissues at Steady-State and During Simian Immunodeficiency Virus Infection
Source: Front Immunol. 2020 Sep 10;11:2134. doi: 10.3389/fimmu.2020.02134 (PMC7511519; doi:10.3389/fimmu.2020.02134)
Supplement: FIGURE S1 — Gating strategy used to define NK cell subsets in the NHP. [file Data_Sheet_1.pdf]

Supplementary Figure 1

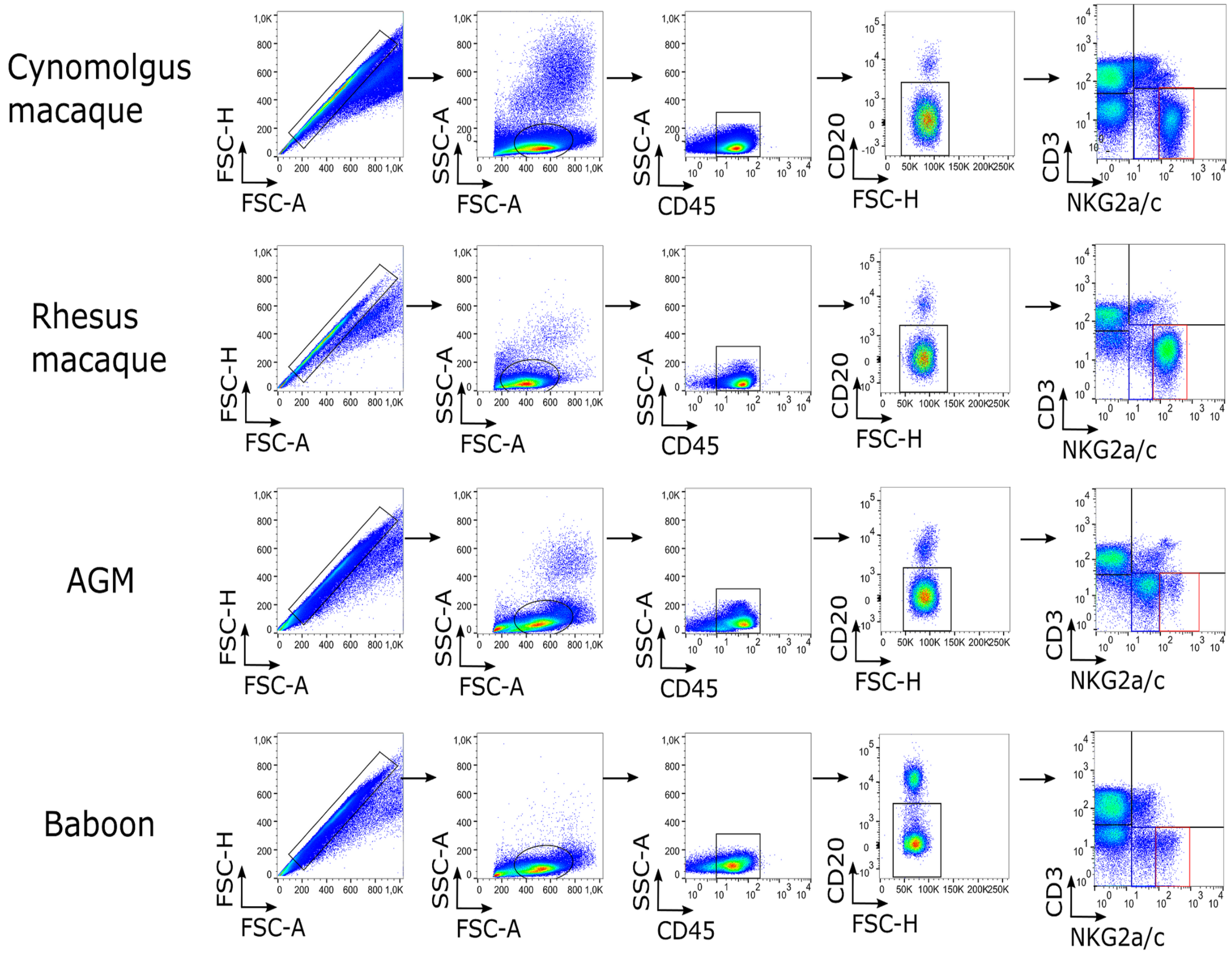

Supplemental Figure 1: main step of the gating strategy used to analyze NK cells defined as CD45$^+$CD3$^-$CD14$^-$CD20$^-$ NKG2$_{a/c}^+$ cells. Representative plots are shown as they were used to identify corresponding cells.

Supplementary Figure 2

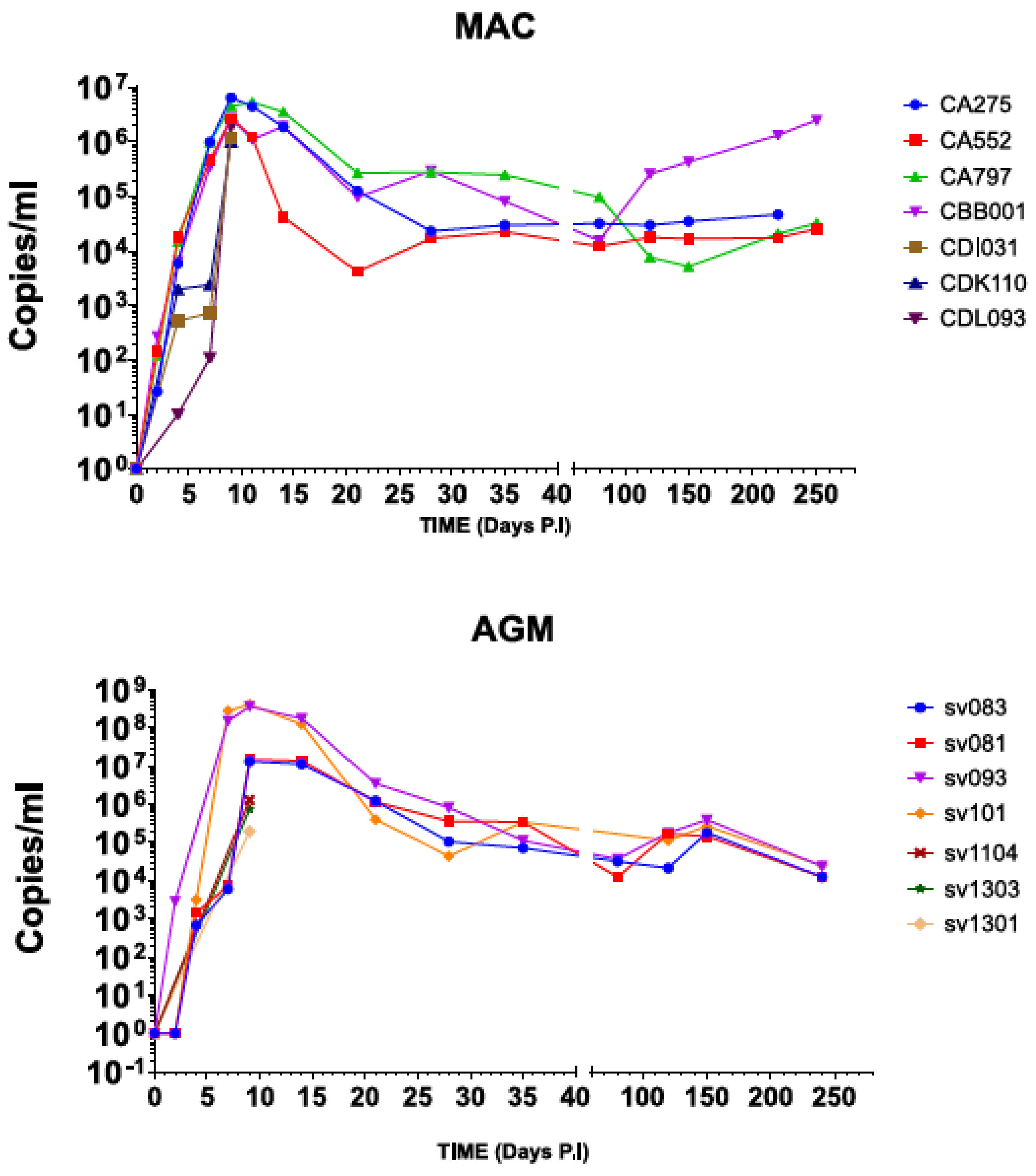

Supplemental Figure 2: Viremia profiles during SIV infections. Seven CM and seven AGM were respectively infected with SIVmac251 and SIVagm.sab92018. The SIV copy numbers in the plasma over time are indicated.Supplementary Table 1

Supplementary Table 1

Antibodies used for flow cytometry.

Antibody	Clone	Suppliers	Control	Reference for Cross reactivity
CD3	SP34-2	BD biosciences	Isotype + FMO	Supplier and ref [1–5]
CD45	D058-1283	BD biosciences	Isotype + FMO	Supplier and ref[4,5]
CD20	2H7	Biolegend	Isotype + FMO	Supplier and ref[5–9]
NKG2A/C	Z199	Beckman Coulter, Inc.	Isotype + FMO	Supplier and ref[5,6,10]
CD16	3G8	Beckman Coulter, Inc.	Isotype + FMO	Supplier and ref[5,6,9,10]
CXCR5	MU5UBEE	Thermo	Isotype + FMO	Supplier and ref[11]
CD8	BW135/80	Miltenyi	Isotype + FMO	Supplier
CXCR3	1C6/CXCR3	BD biosciences	Isotype + FMO	Supplier and ref[5,7,11,12]
NKP30	AF29-4D12	Miltenyi	Isotype + FMO	Supplier
CD69	FN50	BD biosciences	Isotype + FMO	Supplier and ref[3,12]
Nkp46	BAB281	Beckman Coulter, Inc.	Isotype + FMO	Supplier and ref[10]
NKP44	REA1163	Miltenyi	Isotype + FMO	Supplier
CD279(PD-1)	EH12.2H7	BD biosciences	Isotype + FMO	Supplier and ref [11]
Interferon gamma	4S.B3	BD biosciences	Isotype + FMO	Supplier and ref[6,13]
Granzyme B	GB11	BD biosciences	Isotype + FMO	Supplier and ref[6]
TNF-α	MAB11	BD biosciences	Isotype + FMO	Supplier and ref[2,3,6,13]
CD14	Tük4	Miltenyi	Isotype + FMO	Supplier [15]

1. Nehete, P. N., Nehete, B. P., Chitta, S., Williams, L. E. & Abee, C. R. Phenotypic and Functional Characterization of Peripheral Blood Lymphocytes from Various Age- and Sex-Specific Groups of Owl Monkeys (Aotus nancymaae). *Comp. Med.* **67**, 67–78 (2017).
2. Foulds, K. E., Donaldson, M. & Roederer, M. OMIP-005: Quality and phenotype of antigen-responsive rhesus macaque T cells. *Cytometry A* **81A**, 360–361 (2012).
3. Sylwester, A. W., Hansen, S. G. & Picker, L. J. Quantification of T Cell Antigen-specific Memory Responses in Rhesus Macaques, Using Cytokine Flow Cytometry (CFC, also Known as ICS and ICCS): from Assay Set-up to Data Acquisition. *Bio-Protoc.* **4**, e1110–e1110 (2014).
4. Gaufin, T. *et al.* Experimental depletion of CD8+ cells in acutely SIVagm-Infected African Green Monkeys results in increased viral replication. *Retrovirology* **7**, 42 (2010).
5. Huot, N. *et al.* Natural killer cells migrate into and control simian immunodeficiency virus replication in lymph node follicles in African green monkeys. *Nat. Med.* **23**, 1277–1286 (2017).
6. Weisgrau, K. L., Ries, M., Pomplun, N., Evans, D. T. & Rakasz, E. G. OMIP-035: Functional analysis of natural killer cell subsets in macaques. *Cytometry A* **89**, 799–802 (2016).
7. Palgen, J.-L. *et al.* NK cell immune responses differ after prime and boost vaccination. *J. Leukoc. Biol.* **105**, 1055–1073 (2019).
8. Amos, J. D. *et al.* Rapid Development of gp120-Focused Neutralizing B Cell Responses during Acute Simian Immunodeficiency Virus Infection of African Green Monkeys. *J. Virol.* **89**, 9485–9498 (2015).
9. Sugimoto, C. *et al.* Differentiation Kinetics of Blood Monocytes and Dendritic Cells in Macaques: Insights to Understanding Human Myeloid Cell Development. *J. Immunol. Baltim. Md 1950* **195**, (2015).
10. Mavilio, D. *et al.* Identification of NKG2A and NKp80 as specific natural killer cell markers in rhesus and pigtailed monkeys. *Blood* **106**, 1718–1725 (2005).
11. Donaldson, M. M., Kao, S.-F. & Foulds, K. E. OMIP-052: An 18-Color Panel for Measuring Th1, Th2, Th17, and Tfh Responses in Rhesus Macaques. *Cytometry A* **95**, 261–263 (2019).
12. Jacquelin, B. *et al.* Innate immune responses and rapid control of inflammation in African green monkeys treated or not with interferon-alpha during primary SIVagm infection. *PLoS Pathog.* **10**, e1004241 (2014).
13. Hassan, W. M. *et al.* Multivariate profiling of African green monkey and rhesus macaque T lymphocytes. *Sci. Rep.* **9**, 4834 (2019).
14. Schafer, J. L., Li, H., Evans, T. I., Estes, J. D. & Reeves, R. K. Accumulation of Cytotoxic CD16+ NK Cells in Simian Immunodeficiency Virus-Infected Lymph Nodes Associated with In Situ Differentiation and Functional Anergy. *J. Virol.* **89**, 6887–6894 (2015).
15. Diop OM, Ploquin MJ, Mortara L, Faye A, Jacquelin B, Kunkel D, Lebon P, Butor C, Hosmalin A, Barré-Sinoussi F, Müller-Trutwin MC. Plasmacytoid dendritic cell dynamics and alpha interferon production during Simian immunodeficiency virus infection with a nonpathogenic outcome. J Virol. 2008 Jun;82(11):5145-52. Doi: 10.1128/JVI.02433-07. Epub 2008 Apr 2. PMID: 18385227; PMCID: PMC2395206.
